# Plasticity and modulation of olfactory circuits in insects

**DOI:** 10.1007/s00441-020-03329-z

**Published:** 2020-12-04

**Authors:** Sylvia Anton, Wolfgang Rössler

**Affiliations:** 1 IGEPP, INRAE, Institut Agro, Univ Rennes, INRAE, 49045 Angers, France; 2 Behavioral Physiology and Sociobiology (Zoology II), Biozentrum, University of Würzburg, Am Hubland, 97074 Würzburg, Germany

**Keywords:** Antenna, Antennal lobe, Mushroom body, Neuromodulation, Structural synaptic plasticity

## Abstract

Olfactory circuits change structurally and physiologically during development and adult life. This allows insects to respond to olfactory cues in an appropriate and adaptive way according to their physiological and behavioral state, and to adapt to their specific abiotic and biotic natural environment. We highlight here findings on olfactory plasticity and modulation in various model and non-model insects with an emphasis on moths and social Hymenoptera. Different categories of plasticity occur in the olfactory systems of insects. One type relates to the reproductive or feeding state, as well as to adult age. Another type of plasticity is context-dependent and includes influences of the immediate sensory and abiotic environment, but also environmental conditions during postembryonic development, periods of adult behavioral maturation, and short- and long-term sensory experience. Finally, plasticity in olfactory circuits is linked to associative learning and memory formation. The vast majority of the available literature summarized here deals with plasticity in primary and secondary olfactory brain centers, but also peripheral modulation is treated. The described molecular, physiological, and structural neuronal changes occur under the influence of neuromodulators such as biogenic amines, neuropeptides, and hormones, but the mechanisms through which they act are only beginning to be analyzed.

## Introduction

Many insect species predominantly rely on olfaction for intra- and interspecific communication and searching food. Olfaction is of particularly high importance in night- or dim-light active species and for social communication as in social insects. The multitude of available olfactory cues in the natural environment combined with limited size of the nervous system and the resulting neuronal processing capacities render neuronal plasticity and modulation as major factors to optimize the use of neural substrate (Dukas 2008; Gadenne et al. 2016; Groh and Rössler 2020). However, the complexity of the nervous system can also set limits for behavioral and ecological plasticity (Bernays 2001). This finally promotes fitness of an insect in a given ecological and evolutionary context (Agrawal 2001).

Plasticity in insect olfactory systems occurs at multiple levels, for example as a function of physiological state, in response to environmental factors, social interactions, and experience. Whereas most of the literature on the mechanisms of olfactory plasticity and modulation in insects concentrated on the central nervous system (CNS), recent work has also shown modulation already at the olfactory receptor level within olfactory sensory neurons (OSNs) on the antennae, for example, as a function of odor exposure (Tsitoura and Iatrou 2016; Guo et al. 2017; Wicher 2018). Furthermore, modulation of OSN sensitivity due to experience has been described, for example, in male moths (Guerrieri et al. 2012) and due to feeding and maturity in female mosquitoes and blood-feeding bugs (Gadenne et al. 2016 and references therein (Davis 1984; Grant and O’Connell 2007; Siju et al. 2008; Reisenman 2014)). Within the CNS, several levels of plasticity have been identified. We will summarize here mainly the most recent results from studies on plasticity and modulation within the primary and secondary olfactory centers in the brain—the antennal lobes (ALs) and the mushroom bodies (MBs).

Similar to other sensory systems, various mechanisms are involved in olfactory plasticity, and recent methodological advances provide increasing access to study these mechanisms. At the molecular level, the expression of genes associated with olfactory reception and genes coding for neuromodulators and hormones and their receptors can vary in status- or context-dependent manners (Gadenne et al. 2016). At the cellular level, neuronal elements have been identified physiologically and anatomically, and various parameters of their activity were monitored to show modulation (e.g., Neupert et al. 2018). New technology allows to detect the presence of neuromodulators within individual neurons or small populations of neurons, such as biogenic amines, neuropeptides, and hormones (Ly et al. 2019).

Mechanisms of olfactory plasticity have specifically been studied in *Drosophila melanogaster*, due to the available genetic tools. As literature on *D. melanogaster* has been recently reviewed (e.g., Sayin et al. 2018; Amin and Lin 2019; Boto et al. 2020), we concentrate here on other experimental insect models, mainly moths and social Hymenoptera, with a focus on their specific ecological context, because a vast amount of literature is available in these two classical models for olfactory plasticity. We do, however, also include occasional references to further insect species such as locusts, blood-feeding insects, and aphids, because we would like to emphasize and promote the importance of comparative investigations in this field. In order to illustrate neuronal mechanisms, we will provide here an integrative view of olfactory plasticity in a behavioral and ecological context emphasizing the importance of structural neuronal plasticity and neuromodulation in olfaction.

## State-dependent plasticity and modulation

Responses to intra- and interspecific volatile olfactory stimuli can be modulated as a function of the physiological state. Depending on the role of an olfactory cue or signal, the age, reproductive or feeding state, but also the circadian rhythm can influence the sensitivity of the olfactory system to certain olfactory stimuli. Such modulation is mostly caused by an interplay between hormones, neuropeptides, and biogenic amines, acting at the peripheral or the central olfactory levels (for review see Gadenne et al. (2016)). As an example, the titer of the biogenic amine serotonin (5HT) within the AL varies in a circadian fashion in male moths, which is correlated with AL neuron and behavioral responsiveness to sex pheromone or host plant volatiles (Kloppenburg et al. 1999; Gatellier et al. 2004). However, circadian modulation of olfactory sensitivity seems to be primarily modulated at the peripheral level and has been reviewed earlier (Gadenne et al. 2016).

### Reproductive state

The reproductive state, i.e., either the mating state or the capacity to reproduce, has important effects on the responses to pheromones or volatile host cues. In the male moth *Agrotis ipsilon*, behavioral responses to the female-emitted sex pheromone are inhibited transiently after mating. This plasticity seems to originate from a decrease in sensitivity of AL neurons to the sex pheromone, probably through the implication of ecdysteroids, whereas antennal detection of the sex pheromone does not change after mating (for review see Gadenne et al. (2016)). More recently, differences in the occurrence of a few neuropeptides, such as insulin-like peptides, have been found between brains and more specifically ALs of mated and unmated male *A. ipsilon*, indicating a potential role in post-mating sex pheromone response inhibition (Diesner et al. 2018). In another noctuid moth, *Spodoptera littoralis*, behavioral inhibition of male sex pheromone responses after mating rather originates from modulation in OSNs (Kromann et al. 2014). In the same species, an increase in antennal sensitivity to host plant volatiles has been shown in females after mating (Martel et al. 2009; Saveer et al. 2012). Mating-dependent plasticity of peripheral sensitivity to fruit odors and sex pheromones has also been investigated in different fruit fly species. In female *Drosophila suzukii*, mating causes strong up- and downregulation of olfactory genes within the antenna. In parallel, female antennae increased their sensitivity to isoamyl acetate significantly after mating, which is coherent with the attractant role of this compound emitted by fresh fruit to mated females (Revadi et al. 2015; Crava et al. 2019). In *Ceratitis capitata*, sensitivity of antennae and palps to pheromone components emitted by sexually mature males, which attract both males and females, decreases after mating in both sexes (Sollai et al. 2018). However, the mechanisms leading to mating-induced changes in antennal sensitivity are unknown so far. In the hymenopteran parasitoid wasp *Nasonia vitripennis,* females are attracted to a male-emitted sex pheromone, and the mating-induced lack of behavioral pheromone responses seems to be mediated by dopamine, as virgin females injected with dopamine did not respond any more to the pheromone and mated females injected with a dopamine antagonist continued to respond (Lenschow et al. 2018). However, appetitive learning leads to recovery of sex pheromone attraction in these females (Lenschow et al. 2018). Concerning changes in olfactory sensitivity depending on the reproductive state in social insects, very little information is available. In the ant *Harpegnathos saltator*, a reduction in antennal sensitivity to queen-produced cuticular hydrocarbons, involved in inhibiting workers from reproduction, has been revealed in female workers becoming reproductive substitute queens called gamergates (Ghaninia et al. 2017). However, this represents a special case within social Hymenoptera that may be limited to ponerine ants in which mature workers retain the potential to mate and reproduce.

### Feeding state

The effect of the feeding state on olfactory sensitivity has mainly been investigated in blood-feeding insects. The expression of olfactory genes in the antennae and responses of OSNs are modulated after a blood meal in, e.g., mosquitoes, tsetse flies, and triatomine bugs. This leads to reduced behavioral responses to host cues, but increased responses to alternative signals, such as odors emitted by oviposition sites, or aggregation pheromones in bugs (e.g., Rinker et al. 2013; Taparia et al. 2017) (for review see also Gadenne et al. (2016)). Recently, also a role of neuropeptides in the brain has been revealed to contribute to feeding-dependent modulation of olfactory sensitivity in blood-feeding and herbivorous diptera. In the mosquito *Aedes aegypti,* the abundance of two peptides within the ALs, short neuropeptide F2 (sNPF-2) and allatostatin-A-5 (AstA-5), increased 24 and 48 h after a blood meal and systemic injection of both neuropeptides mimicked the host-seeking inhibition effect of a blood meal in unfed females, thus downregulating responses to food odor (Christ et al. 2017). In the oriental fruit fly, *Bactrocera dorsalis*, sNPF has been shown to be involved in feeding state-dependent antennal sensitivity to a host plant odor, but in this case up-modulating responses to food odor. When sNPF gene expression was inhibited via RNAi, the sensitivity to the odor decreased in starved flies, which normally exhibit a high sensitivity (Jiang et al. 2017). In *D. melanogaster*, sNPF also contributes to starving-induced improved responses to food odor at the receptor neuron level and changes odor representation in the AL, resulting in more robust food-search behavior (Root et al. 2011). A more general nutritional effect on olfactory sensitivity has been identified in *D. melanogaster*: when fed with a high fat diet, antennal sensitivity to various odors decreased. This was correlated with a decreased expression of the olfactory co-receptor Orco (Jung et al. 2018). A big challenge now is to unravel how peripheral and central nervous modulation interact in feeding state-dependent changes of olfactory sensitivity.

## Plasticity and modulation related to age and environmental conditions during development

### Environmental conditions during development in non-social insects

Several insect species change their lifestyle and phenotype depending on environmental conditions during postembryonic development and accordingly modify their olfactory communication skills. Locusts, for example, strongly change their lifestyle as a function of population densities during development. When densities exceed a certain threshold, the insects change from a solitary to a gregarious lifestyle (Simpson and Sword 2008). This phase change causes not only behavioral but also morphological and physiological modifications. Among others, the olfactory system is strongly modified: gregarious locusts have less olfactory sensilla on the antennae than solitary locusts, along with a lower discrimination ability for food sources (Greenwood and Chapman 1984; Ochieng et al. 1998). This correlates with a smaller relative size of the ALs in relation to the volume of the entire brain and to the midbrain in gregarious compared to solitary locusts, even though the total brain size is much larger in gregarious locusts (Ott and Rogers 2010). Whereas the anatomy of AL projection neurons did not show any obvious differences between the two phases, solitary adult females possessed a higher proportion of AL neurons responding to two components of the egg-laying aggregation pheromone (Anton et al. 2002). In addition, AL neurons in solitary third instar nymphs responded more frequently to phenylacetonitril, the major component of the adult aggregation pheromone (Ignell et al. 1999). Interestingly, opposing attraction (gregarious) and repulsion (solitary) behavior of the aggregation pheromone are mediated by octopamine and tyramine, respectively (Ma et al. 2015). Phase switch in locusts does not only modify the olfactory system and its sensitivity but also influences associative food odor learning. Gregarious locusts do not acquire new olfactory aversions, contrary to solitary locusts (Simoes et al. 2016).

Many aphid species change their dispersal capacities by producing winged morphs when population density increases, plant quality decreases, or stress factors such as enemy attacks occur (Braendle et al. 2006). The formation of wings in parthenogenetic aphid females improves their dispersal capacities and allows them to colonize new habitats more easily than wingless females. It is known for several aphid species that the sensory equipment of winged individuals is more elaborate than that of wingless aphids: besides differences in eye morphology (Ishikawa and Miura 2007; Kollmann et al. 2010), they possess longer antennae and more olfactory organs, so-called rhinaria, on their antennae (Shambaugh et al. 1978; Miyazaki 1987). A recent study has found evidence that also primary sensory centers in the brain, i.e., visual neuropils and ALs, are larger in winged females than in wingless individuals of the pea aphid, *Acyrthosiphon pisum* (Gadenne et al. 2019). The available genome for this aphid species should allow in the future to pinpoint neuromodulators and their receptors involved in the structural (and probably physiological) changes between winged and wingless females.

### Variations of postembryonic brood care in social insects

Cooperative brood care is a hallmark feature of insect societies, and differential conditions during postembryonic brood development may affect the adult phenotype (Weaver 1957). For example, in the honeybee, the reproductive status and development of the female castes (queen-worker polymorphism) are induced by differential larval feeding and mediated via an epigenetic mechanism involving royal jelly produced in the hypopharyngeal glands (Kucharski et al. 2008). Queens develop from fertilized eggs that are genetically not different from those that develop into workers, but they develop faster, are larger, live much longer, and differ markedly in their adult behavior, including olfactory-guided behaviors. For example, honeybee queens do not respond to their own mandibular pheromone bouquet, and in sterile workers, the response to the queen pheromone is both age- and stage-dependent (Vergoz et al. 2009). Interestingly, the effects of queen mandibular pheromone are mediated by a single component (homovanillyl alcohol) that has high chemical similarity with dopamine and acts on brain dopamine receptors that modulate aversive olfactory learning (Vergoz et al. 2009). The postembryonic pupal development in the two female castes shows marked differences. The ALs develop much faster (by about 4 days) in queens compared to workers, and the same applies to synaptogenesis in olfactory sub-regions within secondary olfactory centers in the MBs (Groh and Rössler 2011). Whereas the number of olfactory glomeruli in the adult AL is only slightly smaller in queens, the spatial arrangement and sizes of individual glomeruli show marked differences in queens compared to workers. Differences in the AL and MB phenotypes are even more pronounced in ants comprising permanent worker castes, especially in leaf-cutting ants (Kelber et al. 2010; Groh et al. 2014). For example, in *Atta vollenweideri,* the development of trail pheromone–specific macroglomeruli is worker size-dependent, and the overall number of glomeruli in a specific AL glomerular cluster (T4 cluster) may differ by more than 50 glomeruli in minor vs. major workers (Kelber et al. 2010). Whether this marked AL polyphenism in the female worker castes is a sole effect of differential feeding still needs to be investigated. Whereas minor workers engage as fungus gardeners inside the nest, large workers leave the nest as foragers and search for profitable food sources using primarily olfactory cues. Consequently, the behavioral responses to trail pheromone were also shown to be worker size-dependent (Kleineidam et al. 2007).

In addition to controlled feeding of larvae, many social insect species provide controlled climate conditions (reviewed by, e.g., Seeley and Heinrich (1981)), which have consequences for metamorphic development including the formation of olfactory centers in the brain. Experimental manipulations in the honeybee have shown that accurate temperature control is required for proper development of olfactory sub-regions in the MBs (Groh et al. 2004, 2006). Slight deviations (1 °C) from the optimal temperature range (36 °C ± 0.5 °C) lead to deficits in synaptic maturation in olfactory input regions of the MBs. The resulting synaptic changes correlate with inferior olfactory learning and memory capabilities or changes in the timing of foraging (Tautz et al. 2003; Jones et al. 2005; Becher et al. 2009). Furthermore, bees raised at lower temperatures performed less well in associative olfactory memory tasks, and they differed in dance-communication performance and undertaking behavior compared to bees raised at higher temperatures within the range of naturally occurring temperatures in the brood area. Similarly, in *Camponotus* ants, workers control the temperature of pupae to specific temperature ranges during postembryonic metamorphic development by brood carrying behavior. Ant nurses respond to changes in the ambient temperature by placing the brood to nest compartments with the appropriate temperatures following circadian rhythms (Roces and Núñez 1989; Falibene et al. 2016). Also in ants, suboptimal temperature regimes affect proper development of olfactory synapses in the MBs (Falibene et al. 2016). Most interestingly, ants that had experienced diverging brood temperature regimes exhibit differences in their stimulus response thresholds for adult brood carrying behavior, most likely due to changes in sensory thresholds (Weidenmüller et al. 2009). Taken together, differential environmental influences caused by variations of brood care conditions during postembryonic metamorphic development affect olfactory circuits in the brain and have consequences for a range of adult olfaction-related behaviors. The above forms of plasticity, therefore, represent interesting cases of metaplasticity (Abraham 2008), meaning that olfactory plasticity induced by brood care conditions affects adult behavioral plasticity in social insect colonies. However, how exactly changes in olfactory circuits are causally linked to changes in complex olfactory behavior (both at the individual and colony levels) still requires further investigations.

### Adult maturation and polyethism

There is ample evidence that early adult development modulates behavioral, peripheral, and central nervous olfactory sensitivity to pheromones in various insects, but also to non-pheromonal odors. During early adult life, increasing antennal responses to pheromones or kairomones have been shown to correlate, for example, with increased odorant receptor expression in mosquitoes or increased hormone receptor expression in a noctuid moth (Bigot et al. 2012; Bohbot et al. 2013). At the CNS level, the age-dependent modulation of attraction behavior and AL neuron sensitivity to sex or aggregation pheromones in moths and locusts has been shown to depend, among others, on juvenile hormone titers (for review see Gadenne et al. (2016)). In various insects, morphological changes have been observed in primary and secondary olfactory centers associated with age-dependent increases in olfactory sensitivity to specific cues or changes in olfactory learning and memory performance (e.g., Huetteroth and Schachtner 2005; Tomé et al. 2014). These age-dependent changes in olfactory sensitivity, at least in moths, have been shown to be independent of experience. 

Adult behavioral maturation and the associated changes in sensory experience affect the olfactory system in social insects. Various studies revealed substantial effects of sensory experience on the development of the AL particularly morphological aspects of individual olfactory glomeruli and their responses to odorants in the AL of honeybees (Winnington et al. 1996; Jernigan et al. 2020). Calcium-imaging experiments suggest that the odor responsiveness of AL glomeruli in honeybee workers increases during the first days of adult life (Wang et al. 2005). Studies in *D. melanogaster* indicate that activity-related volume increases in olfactory glomeruli are mainly caused by an increase in synaptic density within the glomeruli, most likely mediated via local interneurons (Devaud et al. 2003; Sachse et al. 2007). Some of the related changes in olfactory circuits were assigned to age, but the temporal flexibility of task-related changes in adult behavior (adult polyethism) adds another level of complexity of olfactory plasticity in social insects that needs to be studied in more detail in the future.

In addition to the AL, robust structural changes associated with adult behavioral maturation were observed in olfactory input regions of the MBs, as reported by several studies in social Hymenoptera (e.g., honeybee (Withers et al. 1993; Durst et al. 1994; Fahrbach et al. 1998; Groh et al. 2012; Scholl et al. 2014; Muenz et al. 2015), ants (Gronenberg et al. 1996; Kühn-Bühlmann and Wehner 2006; Stieb et al. 2010, 2012)). The cellular processes underlying these volume changes involve massive outgrowth of Kenyon cell (KC) dendrites and, at the same time, pruning of presynaptic boutons within microglomerular synaptic complexes (Farris et al. 2001; Stieb et al. 2010; Groh et al. 2012; Muenz et al. 2015). Dendritic expansion is the main cause for the volume increase in the MB calyx during the transition from nursing to foraging. The overall result of this structural plasticity is an increase in the olfactory projection neuron to KC synaptic divergence of olfactory circuits by about 33% (the number of KC dendritic profiles forming synapses with one projection neuron bouton; Groh et al. 2012). Pharmacological stimulation suggests that the underlying processes are promoted by activity in muscarinic cholinergic transmission during foraging experience (Ismail et al. 2006). Sensory exposure was also shown to play an important role in this olfactory plasticity in leaf-cutting ants (Falibene et al. 2015). A combined anatomical and patch-clamp study in *D. melanogaster* confirmed that structural plasticity of olfactory MB input synapses is induced by sensory activation (Kremer et al. 2010). Interestingly, aged honeybee queens exhibit an increase in the relation of olfactory versus visual input synapses in the MB calyx (Groh et al. 2006). More recent studies revealed that social experience influences the number of MB olfactory input synapses in worker bees (Cabirol et al. 2017, 2018). However, a major problem with manipulations of the social environment is that too many variables (e.g., pheromonal, tactile, visual) may change at the same time and, in most cases, are difficult to control. This problem, for example, became evident while studying the influence of the primer pheromone ethyl oleate on maturation of the olfactory circuits in the honeybee brain (Muenz et al. 2015). Ethyl oleate is present at high concentrations on the cuticle of experienced foragers, sensed by OSNs on the antennae of nurse bees, processed in the AL (Muenz et al. 2012), and finally causes a delay in adult behavioral maturation (Leoncini et al. 2004). Future cohort experiments using more tightly controlled sensory manipulations and high-resolution anatomical and behavioral analyses are needed to dissect the changes in olfactory circuits caused by differences in sensory or social experience in order to find the mechanisms how they affect adult olfactory behavior (Groh and Rössler 2020). Winter bees (the last generation of bees in fall) might be a valuable model for studying adult olfactory plasticity in the future, as they live much longer than summer bees and start to resume foraging in the next spring after staying in the hive during the entire winter. The winter bee model may help to dissect more clearly effects of age and sensory experience. The molecular mechanisms underlying structural neuronal plasticity of olfactory circuits are still unknown. A gene expression study (Becker et al. 2016) revealed several genes that might be associated with epigenetic regulation of neuronal plasticity during behavioral maturation of the honeybee, and GTPase activities were correlated with the nurse-forager transition (Dobrin and Fahrbach 2012). However, in both cases, it remained unclear how the molecular changes causally link to structural plasticity in olfactory circuits, which opens an important field for future studies. Recent studies on changes in the activity of immediate early genes following odorant exposure are highly promising in this respect (reviewed in Sommerlandt et al. (2019)).

The rather drastic interior-forager transition in social insects correlates with changes in diverse neuromodulators and hormones (Hamilton et al. 2016). For example, variations were found in biogenic amine levels (reviewed in Kamhi and Traniello (2013)), juvenile hormone (Robinson 1987; Bloch et al. 2002; Dolezal et al. 2012), and vitellogenin (e.g., Amdam and Omholt 2003). However, the causal links of these modulators and hormones, especially how they affect specific sensory pathways, including the olfactory pathways, and/or individual behavioral modules, are still discussed controversially (reviewed in Hamilton et al. (2016)). In recent years, studies on social Hymenoptera began to focus on the large and diverse group of neuropeptides as potential modulators of behavioral pattern transitions (Takeuchi et al. 2003; Brockmann et al. 2009; Pratavieira et al. 2014; Schmitt et al. 2015, 2017; Han et al. 2015; Gospocic et al. 2017). For example, in the desert ant *Cataglyphis fortis*, tachykinin was shown to express age- and behavioral state-related changes associated with task transitions (Schmitt et al. 2017). In the ponerine ant *Harpegnathos saltator*, corazonin was identified as an important driver of behavioral changes (e.g., worker-specific hunting behavior) associated with the transition of female workers into reproductive substitute queens (Gospocic et al. 2017). Neuropeptides have a specifically high potential to mediate a variety of specific or highly localized modulatory actions on neuronal circuits associated with different behavioral patterns, as they represent a very large and diverse group of messenger molecules that may act both as neurohormones or neuromodulators (e.g., reviewed by Schoofs et al. (2017); Nässel and Zandawala (2019)). Future localization analyses of stage-specific changes in the spatial distribution of individual neuropeptides within primary and secondary olfactory centers combined with functional analyses using manipulation experiments appear highly promising in understanding the role of neuropeptides in age- and stage-specific plasticity of olfactory behaviors and circuits.

## Context-dependent plasticity and modulation

**Fig. 1:**
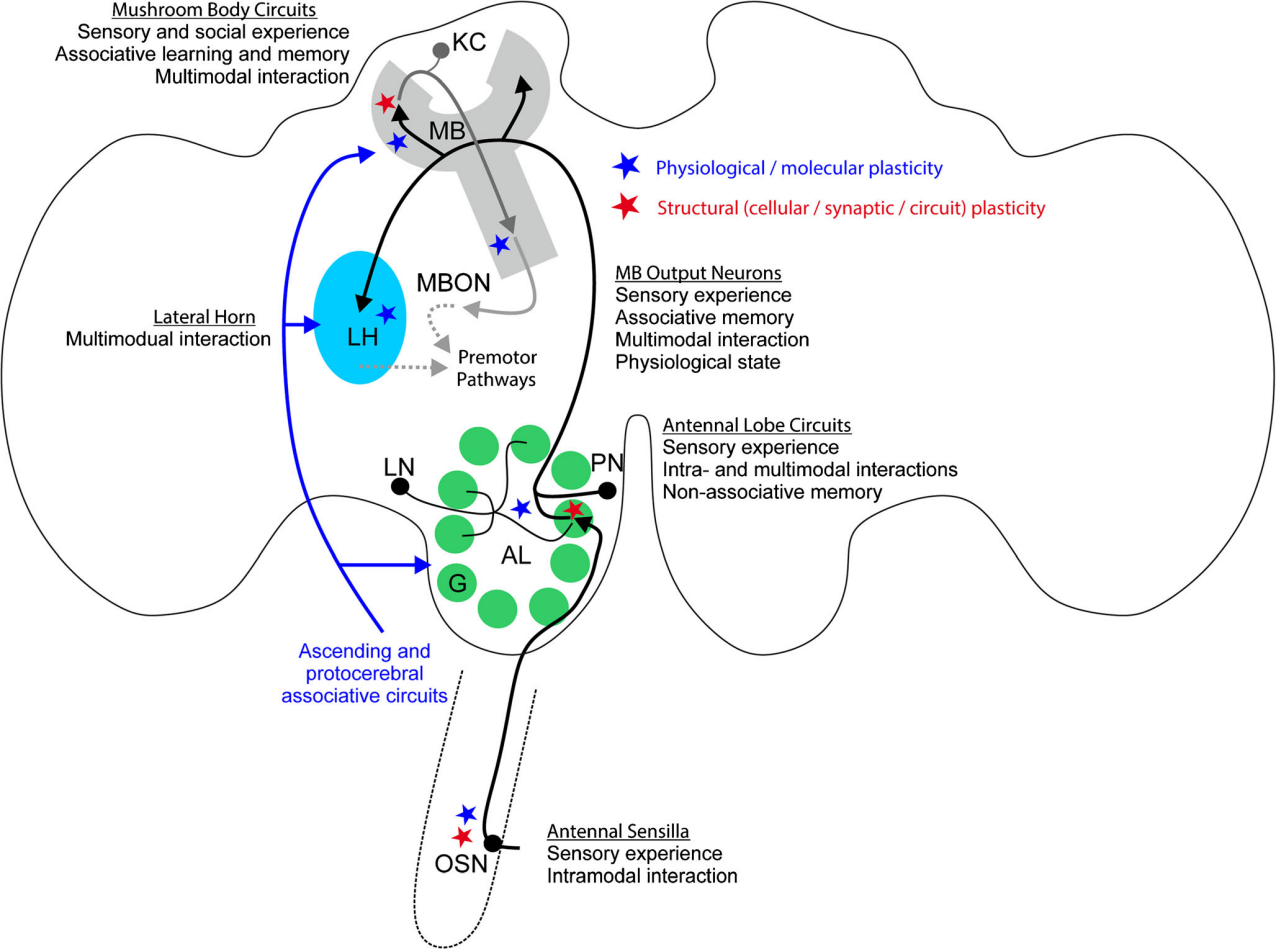
Schematic view of the insect olfactory pathway—from the sensory structures on the antenna to primary (antennal lobe, AL) and secondary olfactory centers (mushroom body, MB and lateral horn, LH) in the brain, indicating factors inducing plasticity and modulation at various processing levels. The blue asterisks indicate sites of action of neuromodulators (biogenic amines, neuropeptides) or hormones and sites for associated physiological and molecular changes (spontaneous activity, response threshold, changes in the expression of odorant receptors, changes in the expression of receptors for modulators or hormones). Red asterisks indicate sites that have been shown to express structural plasticity in olfactory neuronal circuits (structural synaptic changes, changes in axonal/dendritic structure and connectivity, neuropil volume changes). The blue pathway depicts influences of ascending and protocerebral neuronal systems mediating associative influences (e.g., octopaminergic, dopaminergic systems). G, olfactory glomerulus; KC, Kenyon cell; LN, local interneuron; OSN, olfactory sensory neurons; MBON, mushroom body output neuron; PN, projection neuron

Behavioral responses to olfactory signals are modulated by various environmental factors, including different sensory cues emitted by conspecifics, for example, social interactions (see following paragraphs), or other organisms, as well as abiotic factors, such as climate, and pollutants. Such modulation and plasticity can occur at different levels within the olfactory system, starting from the periphery in OSNs, and, in many cases, results in changes within the AL, and especially the MBs (Fig. 1).

### Immediate sensory environment

The presence of different sensory stimuli in the immediate environment of an insect can alter responses to a given olfactory stimulus through interactions of the odorants at the peripheral and/or central level. Even though this type of interaction needs not necessarily fall into the category of modulation, we would like to include them here, because they might interfere or provide the basis for some cases of experience-dependent plasticity. A prominent example is the interaction between sex pheromones and plant-emitted volatiles in male moths. A flower volatile, heptanal, for example, reduces responses to the sex pheromone within the macroglomerular complex of the AL in the noctuid moth *A. ipsilon* both at the input and output level (Deisig et al. 2012) but also results in an improved temporal resolution of pheromone pulses by AL output neurons (Chaffiol et al. 2014). When the two odors are presented with a time shift, the responses of AL neurons to the sex pheromone are delayed as compared to a simultaneous application (Dupuy et al. 2017), which correlates with delayed behavioral responses. In another noctuid moth, *Helicoverpa armigera*, calcium imaging revealed a reduced increase of intracellular calcium levels when stimulated with a blend of sex pheromone and complex plant odors as compared to individual odor application (Ian et al. 2017). On the other hand, synergistic responses to a mixture of sex pheromone and a volatile originating from the larval host plant, pear ester, were reported in the AL of the codling moth *Cydia pomonella* and well correlated with behavioral responses (Trona et al. 2013). In the noctuid moth *S. littoralis*, host plant volatiles enhance the selectivity for conspecific pheromone blends (Borrero-Echeverry et al. 2018), but nothing is known so far about the underlying neural mechanisms.

Non-host volatiles or herbivore feeding-induced volatiles have been shown in several insects to reduce responses to pheromones. One example is the response to aggregation pheromones in bark beetles, which is inhibited by non-host volatiles or volatiles emitted by attacked host trees, originating from inhibition within the OSNs on the antennae (Zhang et al. 1999; Jactel et al. 2001; Andersson et al. 2010). In several moth species, non-host plant volatiles also modulate male sex pheromone responses, but again, interactions have only been investigated at the antennal level (Party et al. 2009, 2013; Faraone et al. 2013; Binyameen et al. 2013; Hatano et al. 2015; Wang et al. 2016). Signals emitted by herbivore-attacked plants can also modulate the attractiveness of host plants for female moths searching for an oviposition site. Females of the tobacco hawk moth, *Manduca sexta*, prefer undamaged host plants above herbivore-damaged plants, in which enhanced emission of (−) linalool renders the signal less attractive (Reisenman et al. 2013). This correlates with inhibitory interactions between two AL glomeruli specific for the two linalool enantiomers in female *M. sexta* (Reisenman 2005). How exactly odor responses are modulated in the presence of other volatiles at the different levels of the olfactory pathway is still a matter of debate. In the peripheral system, direct chemical interactions, competition for binding sites, and interactions within co-localized neurons might be possible (Renou 2014). At the CNS level, additional interactions via separate input channels have to be considered (Renou and Anton 2020).

There is also evidence for the modulation of sex pheromone responses within the AL by mechanical stimulation of the antennae in the noctuid moth *S. littoralis* with a clean air puff (Han et al. 2005). This indicates that modulation of olfactory responses occurs as a function of antennal mechanosensory detection, which can result from air movements in the environment or from feedback of flight activity. Auditory and olfactory inputs also interact in the case of sex pheromone responses in moths when predatory bats emit ultrasound signals. Behavioral responses to the ultrasound signals depend on the quality of the sex pheromone stimulus (Skals et al. 2005). Other sensory modalities, such as vision and taste, are also known to modulate/modify olfactory-guided behavior, but these interactions rather occur within secondary olfactory centers such as the MBs, for example, as shown in moths and honeybees (Balkenius and Balkenius 2016; Strube-Bloss and Rössler 2018).

### Abiotic environmental factors

A major anthropogenic factor influencing the insect olfactory system are insecticides remaining in the environment for a long time. Especially sublethal doses of neonicotinoid insecticides were shown to have negative effects on pollinating insects, such as honeybees and bumblebees, including decreased behavioral responses to attractive olfactory cues and impaired short-term memory, probably due to increased expression of nicotinic acetylcholine receptor expression and increased neural sensitivity to acetylcholine (Desneux et al. 2007; Wright et al. 2015; Cabirol and Haase 2019). Opposite to decreased olfactory responses, sugar sensitivity in honeybees increased after treatments with sublethal doses of the neonicotinoid acetamiprid (El Hassani et al. 2008). Inversely, in the noctuid moth *A. ipsilon*, different sublethal doses of the neonicotinoid insecticide clothianidin were shown to up- or downregulate the sensitivity of AL neurons to the sex pheromone, depending on the dose, and in parallel increased or decreased the behavioral response probability to the sex pheromone (Rabhi et al. 2014, 2016). In another noctuid moth, *S. littoralis*, peripheral and behavioral modulation of sex pheromone responses was caused by sublethal doses of another insecticide, deltamethrin, a widely used pyrethroid (Lalouette et al. 2016). So far, it is, however, not known if the observed modulatory effects of insecticides are caused directly by receptor-ligand interactions, or if insecticides cause modifications of neuromodulator levels or expression of their receptors (Abrieux et al. 2013, 2014, 2016). In addition, pyrethroid insecticides were shown to disturb the wiring of olfactory glomeruli during postembryonic metamorphic development in *M. sexta* (Wegerhoff et al. 2001).

## Olfactory plasticity involving learning and memory

### Non-associative experience

Experience has long been known to modify behavioral responses to olfactory stimuli, and the neuronal and molecular mechanisms underlying these modifications have been investigated for many years. Here we will review only recent data on the role of physiological mechanisms and anatomical long-term modifications that occur within the olfactory pathway as a consequence of different forms of learning in insects. As an extreme case, experience can be acquired during early development and influence larval or adult behavior, or, more frequently, during the adult stage that may lead to long-lasting adaptive changes in olfactory behavior. Even though there are indications, that larval host plant experience in moths modulates female oviposition behavior and even male partner choice, so far the neural substrate concerning the transfer of memories from the larval to the adult stage remains largely speculative (Anderson and Anton 2014).

Simple forms of non-associative experience modulating olfactory-guided behavior, such as sensitization and habituation, have been revealed in many insects. Nevertheless, very little is known about the neural mechanisms underlying these forms of learning. Brief exposure to a behaviorally relevant dose of the sex pheromone in the male moth *S. littoralis*, on the other hand, has been shown to modify the expression of an odorant-binding protein in the antenna and leads to stronger subsequent OSN responses to the same signal (Guerrieri et al. 2012). However, as physiological and anatomical changes occur in the AL, too, upon pheromone exposure, we cannot exclude a feedback to the peripheral system causing this form of sensitization (Anderson et al. 2007; Guerrieri et al. 2012). In addition, brief exposure to various behaviorally active sensory signals, including predator sound and different olfactory stimuli, improved behavioral responses and increased the sensitivity of neurons within the AL to the sex pheromone, rather than in the antennae in the same moth species (Anton et al. 2011; Minoli et al. 2012). At the same time, the volume of the macroglomerular complex (MGC) glomerulus, processing information on the major sex pheromone component, and of the MB calyces increased in size after brief pre-exposure to these same stimuli (Guerrieri et al. 2012; Anton et al. 2015). Attractive and repellent gustatory stimuli also improved subsequent behavioral responses to the sex pheromone, but neither modified AL neuron responses to the sex pheromone nor the volume of MGC glomeruli or the MB calyces, indicating that the behavioral effects might originate from neural modifications in higher brain centers (Minoli et al. 2012; Anton et al. 2015).

### Associative learning and long-term olfactory memory

Associative learning is very common in a large variety of insects. As associative learning and memory represent a very large and rapidly expanding research field, we here mostly focus on plasticity associated with stable olfactory long-term memory (LTM), as it has the potential to affect insect behavior over extended time. The wealth of literature in the field of associative learning and memory, especially in *D. melanogaster*, is beyond the scope of this review (for recent reviews, see, e.g., Kahsai and Zars (2011); Guven-Ozkan and Davis (2014); Sugie et al. (2018); Kacsoh et al. (2019); Boto et al. (2020)). Even though behavioral and molecular studies of learning in parasitoid wasps are numerous (for review see Hoedjes et al. (2011); Smid and Vet (2016)), neurobiological studies besides *D. melanogaster* have largely focused on social insects, which shall be the main topic here. Nevertheless, associative olfactory learning has been evidenced in various other insects, such as moths, locusts, crickets, and parasitic wasps (e.g., Fan et al. (1997); Hartlieb et al. (1999); Daly and Smith (2000); Meiners et al. (2003); Skiri et al. (2005); Costa et al. (2010); Simoes et al. (2016)), but the underlying neurobiological mechanisms are unexplored except for a few rare cases (Cayre et al. 2007; Cassenaer and Laurent 2012). Among the social insects, the honeybee has proven a very valuable model for the study of plasticity related to long-term memory (> 24 h) (e.g., Menzel (1999); Müller (2000); Menzel and Giurfa (2001); Menzel et al. (2007)). Experience-related changes in the activity of glomeruli were described in the AL of the honeybee using calcium-imaging techniques indicating that changes in olfactory responses persist over extended time periods after associative learning at this early sensory processing level (Rath et al. 2011). The robust and well-studied proboscis extension response is a favorable behavioral paradigm for classical conditioning to study olfactory LTM in detail. Using sequential associative conditioning, bees can be trained to memorize the association between a sugar reward and an odor over extended time (> 3 days up to weeks, months, or lifetime). The reward or punishment pathways for appetitive and aversive olfactory learning have been linked to ascending and brain octopaminergic and dopaminergic modulatory systems—mainly via their influences on odor responses at the level of the MBs, but also at the levels of the AL and lateral horn (LH) (Mauelshagen 1993; Hammer and Menzel 1995; Okada et al. 2007; Tedjakumala et al. 2014; Jarriault et al. 2018). Whereas the majority of modifications due to associative LTM are localized at the CNS level, physiological plasticity associated with LTM has nevertheless also been evidenced at the antennal level. Expression of olfactory receptors known to bind the learned odor compounds was significantly downregulated after associative learning, and electroantennogram responses were significantly reduced in honeybees which had formed a LTM, compared to control bees (Claudianos et al. 2014). The feedback mechanism towards the CNS, however, remained unclear in this case. At the CNS level, the formation of a stable olfactory LTM was shown to be transcription-dependent and to involve structural synaptic changes in olfactory circuits at the input of the MBs (Hourcade et al. 2010). Only bees that had received paired stimulation of the conditioned (odor pulse, CS) and unconditioned stimulus (sugar water, US), and that were not injected with the transcription inhibitor actinomycin D (ActD) after training, had retained a stable LTM when they were tested with the CS after 3 days. Most interestingly, stable LTM formation after 3 days was associated with an increase in synaptic complexes within olfactory compartments of the MB calyces. This effect was absent in neighboring visual input regions. Naïve bees, i.e., bees that had received unpaired stimulation and paired stimulated bees that had received ActD, were unable of memory retrieval and did not show any changes in synaptic densities. The authors conclude that growth of new synapses may be involved in stable LTM in the insect brain, similar to what has been found in the mammalian brain (Abraham et al. 2019). Compared with synaptic pruning following sensory exposure as described above, associative olfactory learning and the formation of transcription-dependent stable LTM resulted in a volume-independent increase of synaptic complexes in olfactory compartments of the honey bee MBs (Groh and Rössler 2020). This suggests that the increases in densities of synaptic boutons after associative LTM formation may represent a form of learning-related (Hebbian) structural plasticity in MB-calyx microcircuits. Transcription-independent memories, such as early long-term memory, did not lead to any detectable structural changes in olfactory circuits. Multiple-trial conditioning leading to LTM has previously been shown to depend on intracellular calcium levels, which indicates a role of calcium in structural plasticity associated with stable LTM (Perisse et al. 2009). In the same line (Scholl et al. 2015), using RNAi knockdown and pharmacological manipulation in the MBs showed that CaMKII is required for the formation of both early and late olfactory LTM, indicating that the calcium-dependent “learning protein” might be involved in triggering structural synaptic plasticity. The above studies suggest that olfactory LTM is associated with structural changes in olfactory circuits in the MBs. However, we have to keep in mind that structural changes in olfactory synaptic circuits themselves may be part of a memory trace, but whether they are actually required for memory storage and retrieval remains to be determined.

A similar effect was observed in leaf-cutting ants, in that case after aversive olfactory learning of odors associated with unsuitable plant material for cultivating the underground symbiotic fungus maintained by the ants (Falibene et al. 2015). The formation of an aversive olfactory LTM leads to an increase of the synaptic densities in olfactory (not visual) circuits of the MBs, whereas pure sensory exposure resulted in synaptic pruning. Whereas the increase of synaptic boutons may also represent a form of Hebbian plasticity, pruning of synapses after pure sensory exposure may lead to adjustments in MB circuits resulting in homeostatic regulation to a drastically changing olfactory sensory input.

Physiological access to plasticity of olfactory circuits in the MBs is sparse, except for few calcium-imaging studies suggesting physiological plasticity at the olfactory projection neuron-to-KC synapses and electrophysiological recordings revealing spike-timing-dependent plasticity at mushroom body output neuron synapses (Faber et al. 1999; Szyszka et al. 2008; Cassenaer and Laurent 2012). Learning-related olfactory plasticity was also revealed by intracellular recordings and calcium imaging of GABAergic neurons in the honeybee forming recurrent circuits from the MB output to the input (Grünewald 1999; Haenicke et al. 2018). Similarly, recordings revealed olfactory plasticity in another type of MB extrinsic neurons (Haehnel and Menzel 2012). However, as intracellular recordings and calcium imaging are limited to short-term recording times, it is difficult or rather impossible to monitor changes over extended periods, for example, after associative conditioning. More recent studies employing long-term recordings (over several hours to days) of MB extrinsic or MB output neurons via multiple thin wire tetrodes emerged as a feasible approach to monitor learning- and memory-related long-term changes in olfactory circuits. In the honeybee, multi-unit recordings can even be combined with olfactory conditioning experiments using the proboscis extension response (Strube-Bloss et al. 2012). This technique also opens up possibilities to look into multimodal (olfactory-visual) interactions and their role in context-specific influences on olfactory perception (Strube-Bloss and Rössler 2018).

## Outlook

A major conclusion from previous studies is that plasticity and modulation occur at all levels of the insect olfactory pathway. Whereas some drivers of plasticity like internal programs, age- and status-/stage-specific causes of plasticity, seem to act at both peripheral and central levels, experience-dependent plasticity like learning and memory as well as multimodal interactions preferentially, but not exclusively, occur at higher central levels, particularly the MB. The mechanisms by which sensory and modulatory influences target the different levels of the olfactory pathway are comparably well understood for learning and memory in the MBs (especially from work in *D. melanogaster* and the honeybee on dopaminergic or octopaminergic modulation). Much less, however, is known for other modes of plasticity including bottom-up and top-down influences of olfactory memory. This clearly needs more intense investigations in the future, for example, efforts to understand distributed forms of plasticity and to identify the major neuromodulators, for example, within the large family of neuropeptides. Furthermore, we need to find causal links between changes in gene expression or epigenetic regulation, messenger molecules, and their action on identified neuronal circuits all the way up to how plasticity in these circuits modulates behavior. In addition, we need more information on local modulatory interactions, such as between different olfactory glomeruli in the AL, recurrent pathways within the MBs, or interactions (bottom up and top down) between primary and secondary olfactory centers (MB, LH, and AL). In that respect, MB output neurons (MBONs) might play a key role in mediating such interactions. Integrative and multidisciplinary approaches at different levels are necessary to fully understand the mechanisms underlying age-, status-, and state-specific changes in olfactory processing and perception.

Another important perspective is to investigate the role of multimodal interactions, aiming towards understanding multisensory, context-dependent plasticity influencing olfactory perception. Here the role of the MBs has been highlighted, but the function of other protocerebral neuropils, like the function and potential interactions with lateral horn neurons, is still largely unexplored in most insects. Recent advances in high-resolution insect neuronal brain atlases that started in *D. melanogaster* (Dolan et al. 2019) will help to explore plasticity in these brain areas. The potential role of the lateral horn in memory formation should be explored in future studies, as another recent study in *D. melanogaster* already showed that specifically context-dependent LTM appears to be mediated by lateral horn neurons after only single trial conditioning (Zhao et al. 2019).

In evolutionary terms, variations in olfactory plasticity between different insect species provide a promising source of knowledge to understand their efficient adaptation to the environment. Insects represent by far the most abundant group of animal species with highly diverse lifestyles. Because of this rich species diversity and the multitude of evolutionary adaptations across insect taxa, it will be most important to promote comparative research on plasticity in the olfactory system of diverse insect species. This includes classical model insects like *D. melanogaster,* using the powerful genetic manipulations available, but equally important, non-model insect species should be investigated to reveal insight into novel modes of plasticity in their olfactory systems and behaviors. Further investigations on the two focus groups of this review, moths and social Hymenoptera, with rich knowledge on their olfactory systems, behaviors, and their plasticity, are specifically important from an applied point of view, because they include both important pest species, but also beneficial (pollinator) species. Understanding olfactory plasticity in these insects will largely contribute to efforts of environmentally acceptable control of pest insects and to improve protection of beneficial species. To study non-model insects, novel tools like CRISPR/Cas9 manipulation of gene expression already started to become very helpful. Comparative mechanistic approaches are highly important in future research aimed at understanding the role of olfactory plasticity in the dynamics of adaptation of insect species under global change. Pre-adaptations for high levels of olfactory plasticity may allow species more easily to invade new habitats in the course of climate change. Olfactory plasticity is also an important feature from an ecological point of view. We should investigate how different lifestyles and interactions within trophic networks as well as with the abiotic environment influence plasticity. Studies on the mechanistic nature and role of such differences between closely and distantly related insect species with similar or different lifestyles, habitat preferences, and olfactory behaviors provide a rich ground for future comparative research on the causes and consequences of olfactory plasticity.

There is still a long way to go until we fully understand the powerful mechanisms and influences of olfactory plasticity and modulation on insect behavior and their ecological consequences. Both the experimental accessibility and rich diversity of insects clearly promise exciting future advances in this important field of research.
